# More than numbers: survival, symptoms, and what care triggers

**DOI:** 10.1007/s12471-026-02024-y

**Published:** 2026-01-21

**Authors:** Pim van der Harst

**Affiliations:** Department of Cardiology, University Medical Centre Groningen, Groningen, The Netherlands

In this issue of the *Netherlands Heart Journal*, four original papers present a familiar theme from a different angle: outcomes are rarely a single number, and context matters.

Derks et al. link nationwide claims, registry data and death certificates to clarify all-cause mortality after major cardiac interventions [1]. Among 203,001 procedures, 13.7% died within 5 years. Early deaths are mainly cardiovascular, but after 2 years non-cardiovascular causes dominate. For benchmarking, long-term outcomes increasingly reflect comorbidity and competing risks, so fair comparison requires solid case-mix correction and broader prevention beyond the cath lab.

Oirbans et al. assess HRQoL after pulmonary vein isolation for atrial fibrillation using AFEQT in the Netherlands Heart Registration [2]. Scores improve at 1 year, yet many remain impaired; female sex and baseline symptom burden predict outcomes. Findings guide counselling, expectation-setting, and choice of follow-up or alternatives.

Voorhout et al. address a daily reality: patients with non-typical chest pain discharged from the emergency department. Routine cardiology follow-up within 3 months was linked to more repeat ED visits (13.6% vs 5.1%), more downstream testing, and no reduction in MACCE [3]. Reassurance is not a test, and follow-up may amplify medicalisation rather than reduce uncertainty.

Finally, Peeters et al. show that implementation itself can be an intervention [4]. Updating an acute heart failure protocol increased uptake of evidence-based measures, including acetazolamide and intravenous iron, with a trend toward broader guideline-directed therapy, at the cost of a 1-day longer admission. Simple tools, repetition, and shared routines can translate guidelines into bedside behaviour.

Together, these papers nudge us toward a more mature definition of success: doing the right things for the right patient, measuring what matters over time, and staying honest about where benefits end and unintended consequences begin.
